# Autotaxin Signaling Governs Phenotypic Heterogeneity in Visceral and Parietal Mesothelia

**DOI:** 10.1371/journal.pone.0069712

**Published:** 2013-07-25

**Authors:** Elaine L. Shelton, Cristi L. Galindo, Charles H. Williams, Elise Pfaltzgraff, Charles C. Hong, David M. Bader

**Affiliations:** 1 Department of Medicine, Vanderbilt University, Nashville, Tennessee, United States of America; 2 Department of Cell & Developmental Biology, Vanderbilt University, Nashville, Tennessee, United States of America; 3 Department of Pharmacology, Vanderbilt University, Nashville, Tennessee, United States of America; 4 Research Medicine, Veterans Affairs, Nashville, Tennessee, United States of America; Cincinnati Children's Hospital Medical Center, United States of America

## Abstract

Mesothelia, which cover all coelomic organs and body cavities in vertebrates, perform diverse functions in embryonic and adult life. Yet, mesothelia are traditionally viewed as simple, uniform epithelia. Here we demonstrate distinct differences between visceral and parietal mesothelia, the most basic subdivision of this tissue type, in terms of gene expression, adhesion, migration, and invasion. Gene profiling determined that autotaxin, a secreted lysophospholipase D originally discovered as a tumor cell-motility-stimulating factor, was expressed exclusively in the more motile and invasive visceral mesothelia and at abnormally high levels in mesotheliomas. Gain and loss of function studies demonstrate that autotaxin signaling is indeed a critical factor responsible for phenotypic differences within mesothelia. Furthermore, we demonstrate that known and novel small molecule inhibitors of the autotaxin signaling pathway dramatically blunt migratory and invasive behaviors of aggressive mesotheliomas. Taken together, this study reveals distinct phenotypes within the mesothelial cell lineage, demonstrates that differential autotaxin expression is the molecular underpinning for these differences, and provides a novel target and lead compounds to intervene in invasive mesotheliomas.

## Introduction

Mesothelia cover all coelomic organs and line all body cavities of the vertebrate providing a smooth, lubricated surface on which organs move. While healthy mesothelia in the adult appear uniform and innocuous, this cell type is actually highly plastic in nature performing multiple and varied functions in the embryo and adult. During organogenesis, mesothelia undergo epithelial mesenchymal transition (EMT) to provide all or nearly all vasculogenic cells in the generation of blood vessels as well as producing diverse stromal cells to internal organs [1], [2], [3], [4], [5], [6]. In the adult, mesothelia can undergo EMT generating cells critical in tissue repair while over-population of these cells in scaring and fibrosis in the heart, lung, and abdominal organs presents a major health problem [7], [8], [9], [10]. Finally, the cancerous derivative of mesothelia, mesothelioma, has long been recognized as a highly invasive and pathological cell type [11], [12].

Given the diversity of function in these various settings, it is interesting to note that nothing is known about potential heterogeneity within this cellular phenotype. As exemplified by a recent report [13], mesothelia are considered a singular cell type. In fact, the assumption has been made that all mesothelia are “the same cell” [14], “hardly varying from site to site” [15]. Further, mesothelia are characterized by uniformity in morphology and shared expression of numerous marker genes [16], [17] which has led to the position that all mesothelial cell types are “similar regardless of species or anatomical site [14].” While completely untested, this blanket characterization may be inaccurate. For example, all human skeletal muscle, regardless of anatomical location, share general cellular and structural characteristics. However, variation in gene expression within this cell type results in heterogeneity of fiber types underlying their critical functional diversity [18]. To combat abnormal mesothelial development and disease, it is essential to understand the basic mesothelial phenotype and identify molecular targets for intervention.

Here, using the most basic subdivision of this cell type, visceral and parietal mesothelia, we reveal that mesothelia are not “similar”. In fact, gene profiling and protein expression analyses determined that these two mesothelia have fundamentally different patterns of gene expression. Visceral and parietal cells also exhibit dramatically different phenotypes in terms of such basic properties as movement, adhesion, invasion, and differentiation which define this cell type. Interestingly, autotaxin, a secreted lysophospholipase D that converts lysophosphatidylcholine into the lipid signaling molecule lysophosphatidic acid (LPA) and known to influence the aforementioned properties in other cell types [19], [20], [21], [22], [23], was identified as the most differentially distributed gene product with significantly higher expression levels restricted to the visceral phenotype. Importantly, we find that autotaxin, originally described as a tumor cell-motility-stimulating factor [24], is expressed at abnormally high levels in invasive human mesotheliomas. Using two newly discovered small molecules inhibitors of the downstream mediator LPA, we demonstrate that autotaxin signaling is indeed an essential regulator underlying the differences between these two mesothelial phenotypes and mediates the migratory and invasive behaviors of human mesotheliomas. Importantly, we also show that these new reagents can be used to halt migration and invasion of these cancerous cells. Taken together, this study reveals that autotaxin drives fundamental diversity within mesothelial phenotypes, and describes two novel therapeutic lead compounds to blunt invasiveness of tumors, specifically mesotheliomas.

## Results

### Identification of fundamental differences in mesothelial phenotypes

**Figure 1:**
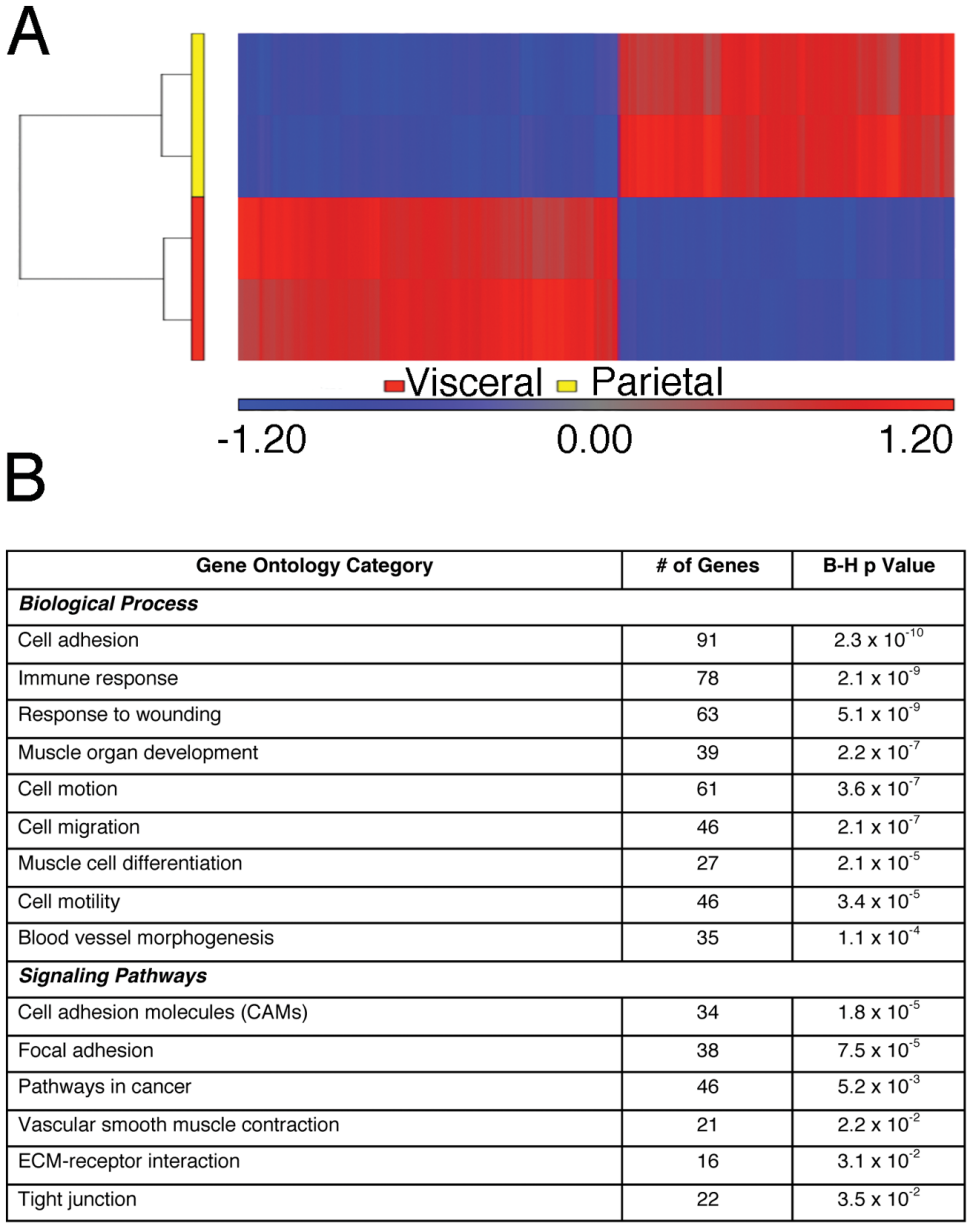
Visceral and parietal mesothelia have significantly different gene expression profiles. Microarray analysis was performed on visceral and parietal mesothelia isolated from adult mice. Hierarchical clustering of 1,728 probes separated the two cell types based on gene expression patterns. Vertical dendrograms represent the individual samples, of which there are two replicates for each tissue type (A). Values shown are log base 2 and bright red, bright blue, and gray indicate the highest, lowest, and median normalized signal values respectively. Overrepresented functional categories were generated for both tissue types (B). B-H p values represent the Benjamini and Hochberg corrected p value, calculated by the online database and functional analysis program, DAVID, using Fisher's Exact test.

To probe for potent variation in mesothelia, visceral (omental) and parietal (body wall) mesothelia were isolated and subjected to microarray analyses. Greater than 95% of input was from mesothelial cells as judged by the eYFP marker expressed in a mesothelial-specific manner in Wt1-cre; Rosa26R^eYFP^ mice. Striking differences in gene expression profiles were immediately obvious between the two phenotypes. Statistical analysis of 1,728 differentially-hybridized probes successfully separated the two cell types based on gene expression patterns (Figure 1A). 796 unique transcripts were more highly expressed in visceral mesothelium, while 633 unique genes were more highly expressed in parietal mesothelium demonstrating the heterogeneity of mesothelial cell types. Based on gene ontological analysis (DAVID; [25]), the most over-represented biological functions in this data set were important in adhesion, migration, wound responses, blood vessel development, muscle cell differentiation, and tumor progression (Figure 1B and Table S1).

**Figure 2:**
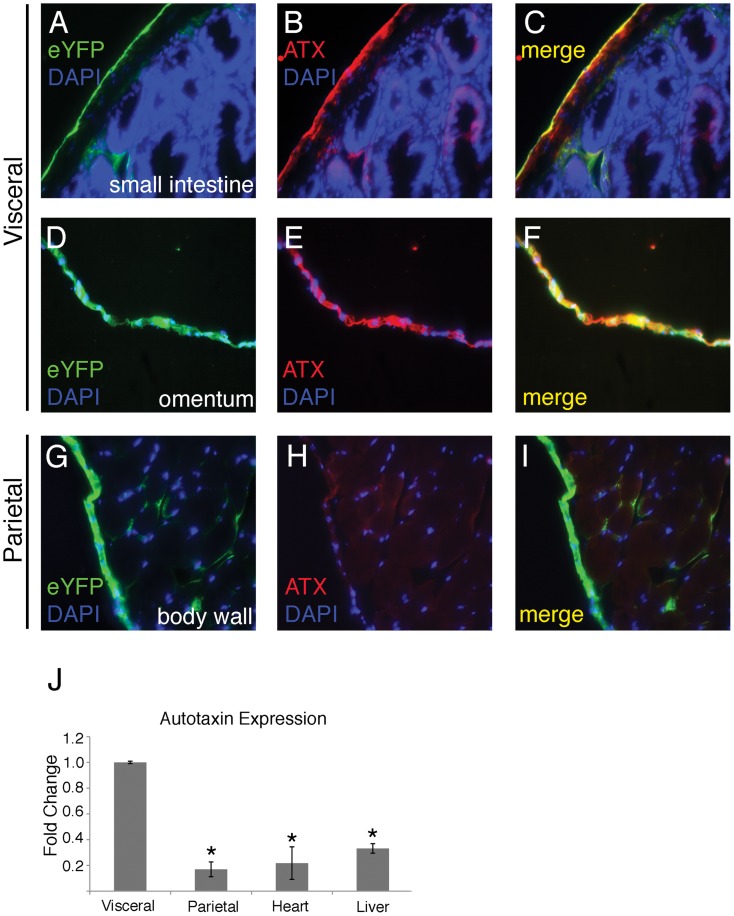
Autotaxin is expressed at significantly higher levels in visceral mesothelia compared to parietal. Immunofluorescence was used to detect autotaxin (ATX) expression in visceral and parietal tissues from Wt1^cre/+^; R26R^eYFP/+^ reporter mice. Cells that express Wt1, a mesothelial marker, will express enhanced yellow fluorescent protein (eYFP) that can be detected with a green fluorescent protein (GFP) antibody (A, D, G). Robust autotaxin expression is evident in visceral mesothelia (A–F). In contrast, the parietal mesothelium that lines the body wall expresses the mesothelial marker (G), but is devoid of autotaxin expression (H). Nuclei are marked with DAPI. Quantitative real-time RT-PCR was used to determine autotaxin transcript levels in visceral (omental) mesothelium, parietal mesothelium, heart, and liver (J). The autotaxin expression in visceral cells was set to 1 and fold changes were calculated for each subsequent tissue type. The asterisk represents a statistically significant difference when compared to the expression level in visceral cells (p<0.05, n = 4). Error bars were calculated using standard error of the mean.

**Figure 3:**
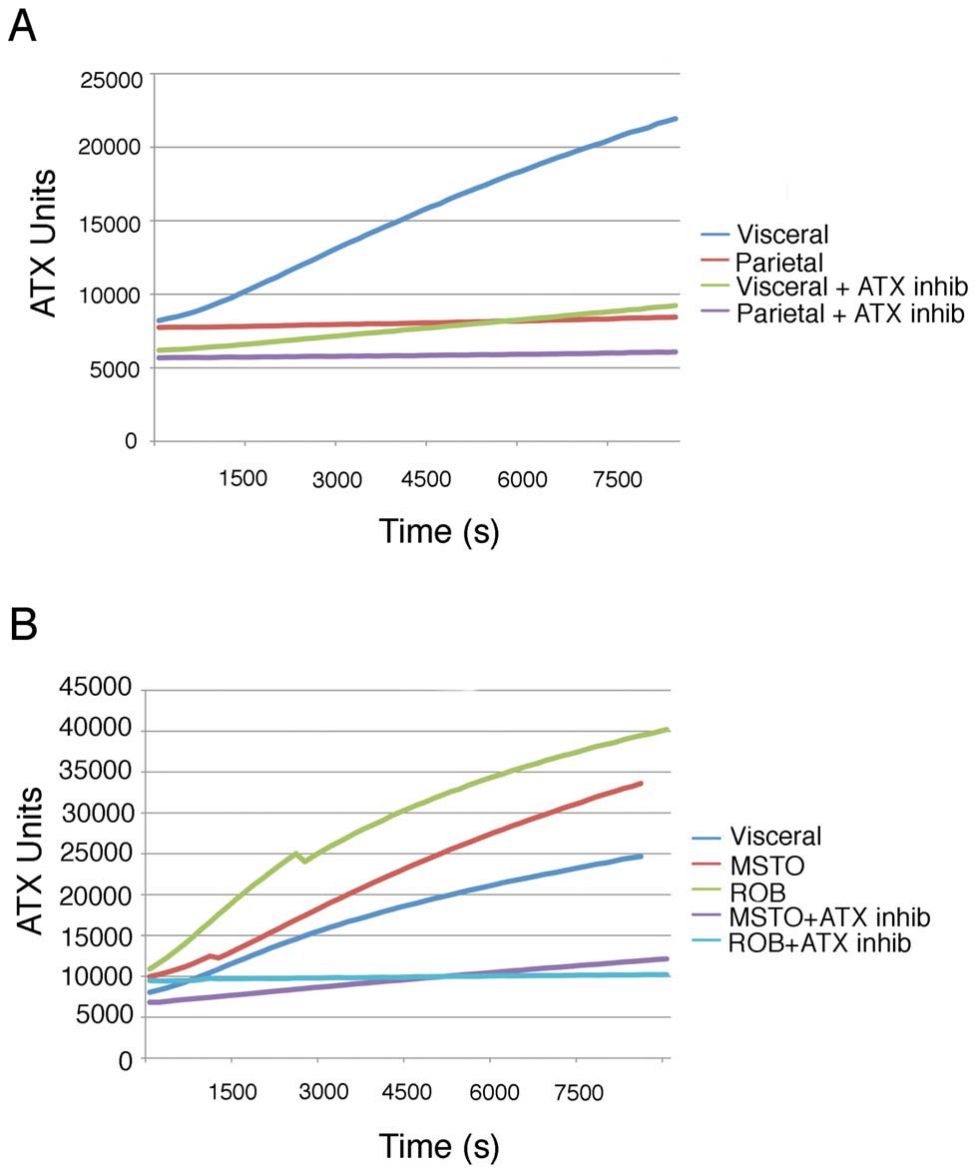
Autotaxin activity is significantly up-regulated in visceral mesothelia and mesotheliomas. Conditioned media was collected from cultured visceral mesothelia, parietal mesothelia, peritoneal mesothelioma (MSTO), and pleural mesothelioma (ROB) cells. Some cultures were treated with the autotaxin inhibitor S32826. Media was assayed for LPA accumulation as a measure of autotaxin activity over a 2 hour period. Visceral mesothelia had significantly higher levels of autotaxin activity compared to parietal cells (A). Furthermore, MSTO and ROB cells had even higher levels of autotaxin activity compared to normal mesothelial cells (B). Normal and pathological cells were responsive to S32826 and displayed decreased levels of autotaxin activity (A–B).

The most differentially expressed gene product was autotaxin (48.4-fold higher expression in visceral mesothelium). This was of particular interest as autotaxin has suggested roles in promoting the aforementioned cellular activities which fundamental to mesothelial function. Detailed IF analysis demonstrated that autotaxin was specifically localized to visceral mesothelium on the surface of the small intestine and the omentum (see colocalization with the transgenic marker, Figure 2A–F). No other cell types of the intestinal wall including smooth muscle, stroma, and mucosal epithelia expressed autotaxin at detectable levels. Interestingly and in agreement with microarray analyses, autotaxin expression was undetectable in eYFP-positive parietal mesothelia (Figure 2G–I). Subjacent body wall tissues were also negative for this protein. Quantitative RT-PCR analysis confirmed that autotaxin was highly expressed in visceral mesothelium (82% increase) compared to its parietal counterpart (Figure 2J). Finally, conditioned media from cultures of isolated visceral and parietal mesothelia were probed for LPA, the conversion product of autotaxin enzymatic activity. Again, visceral cells had a significantly higher level of autotaxin activity (Figure 3A). Addition of autotaxin inhibitor S32826 [26] to visceral cultures dropped LPA production to levels seen in parietal cultures demonstrating the specificity of the assay (Figure 3A). Thus, gene expression profiles of the most basic subdivision of mesothelia are inherently different.

### Visceral and parietal mesothelia are fundamentally different in their ability to adhere, migrate, and invade

Adhesion, migration, and invasion are basic behaviors inherent to mesothelia and distinct differences in these properties may define heretofore unidentified phenotypes within the cell type. To test this hypothesis, visceral and parietal cells were isolated, dissociated into single cells, and probed for potential differences in these basis mesothelial functions.

**Figure 4:**
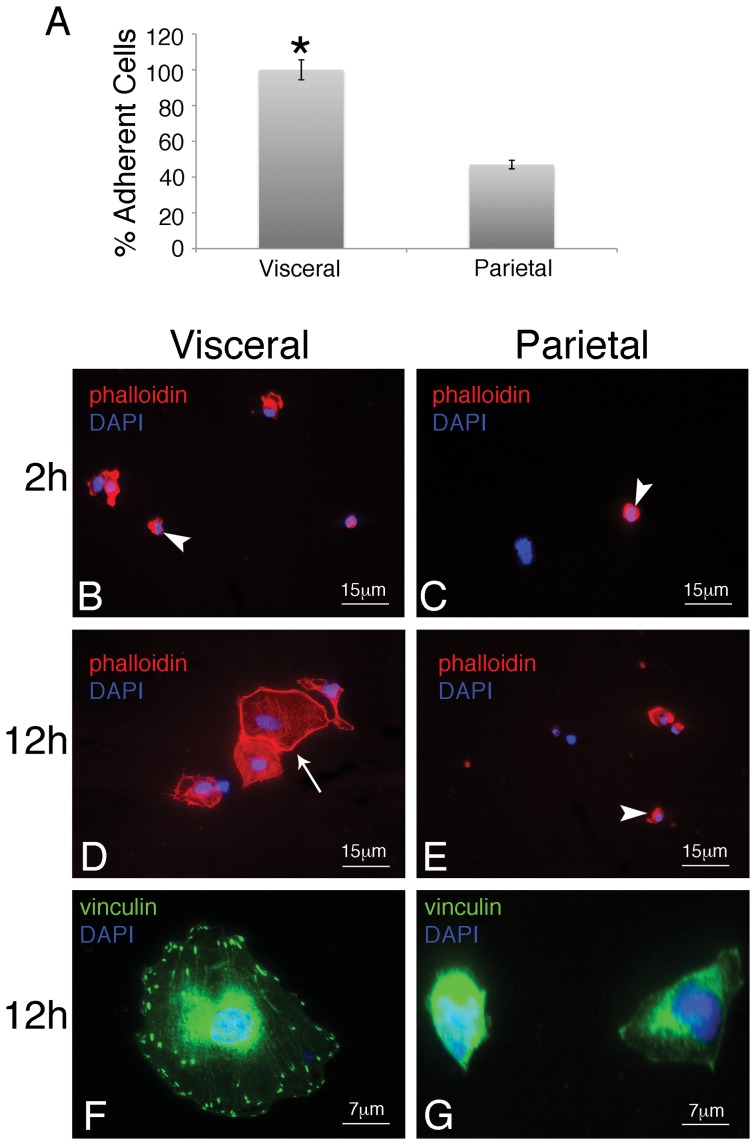
Visceral mesothelial cells are more adherent then their parietal counterparts. Visceral and parietal mesothelia was isolated, dissociated, and plated on fibronectin-coated glass for 2 or 12 hours. After 2 hours, 57% more visceral cells attached to the fibronectin than parietal cells (A). Of the adherent cells, virtually all of the visceral cells had filamentous F-actin expression (B), in contrast to only 50% of the parietal cells. By 12 hours, visceral cells displayed a spread confirmation (D) with prominent vinculin expression at sites of focal adhesion (F). In contrast, parietal cells maintained a round confirmation (E) and had no focal adhesions (G) at this time point. The asterisk represents a statistically significant difference between the percent of adherent visceral and parietal cells (p<0.05, n = 6). Error bars were calculated using standard error of the mean.

Immediate variation in response was detected as visceral cells were highly adherent to fibronectin matrices as compared to the parietal type (Figure 4A). The visceral phenotype displayed a spread conformation with prominent focal adhesions (4F). In contrast, time-matched parietal cells were far less adherent (50% reduction in adherent cells) and remained round in morphology completely lacking focal adhesions (Figure 4E and G).

**Figure 5:**
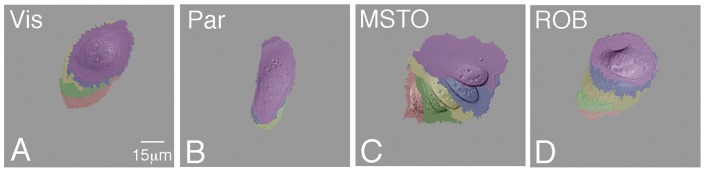
Mesothelia have varying abilities to migrate. Visceral mesothelial (A), parietal mesothelial (B), pleural mesothelioma (C), and peritoneal mesothelioma (D) cells were plated on fibronectin and subjected to time-lapse imaging in order to visualize their ability to migrate. Panels A–D are compilations of static images taken at 20 minute intervals. False coloration indicates a cell's location at each interval: red 0 min; green 20 min; yellow 40 min; blue 60 min; purple 80 min.

**Figure 6:**
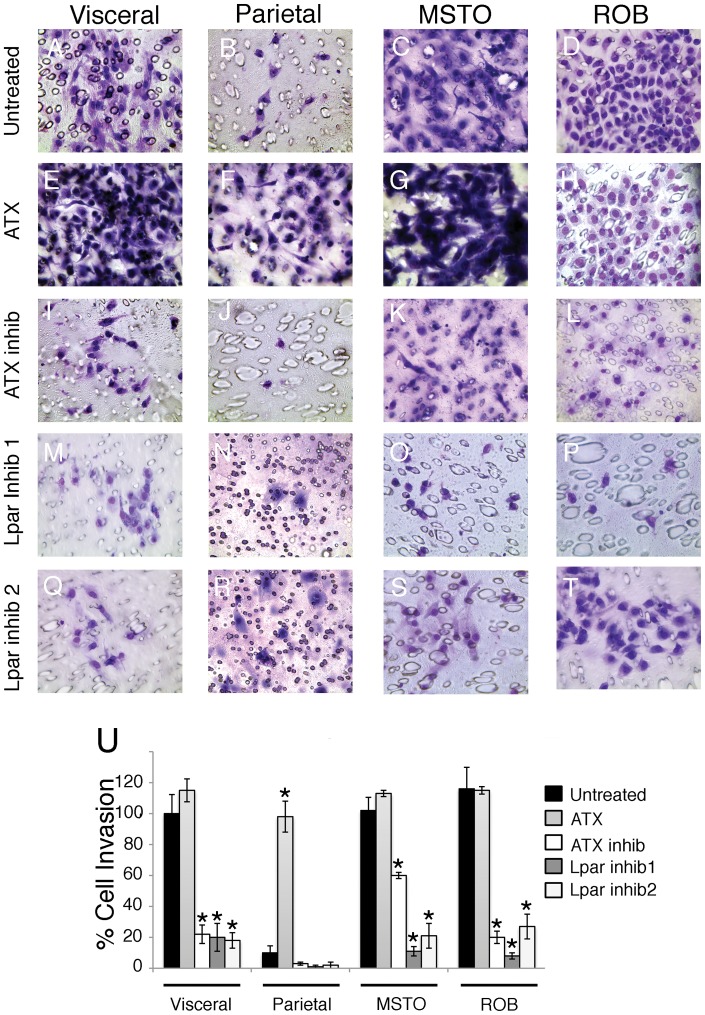
Autotaxin signaling regulates mesothelial and mesothelioma cell invasion. Visceral mesothelial, parietal mesothelial, pleural mesothelioma (MSTO), and peritoneal mesothelioma (ROB) cells were cultured on transwell filters with and without addition of autotaxin (ATX), the autotaxin inhibitor S32826, Lpar1 inhibitor 1 (2440), or Lpar1 inhibitor 2 (8437). Of all the cells studied, the mesothelioma cells were the most invasive (C, D). Visceral cells (A) invaded more readily than parietal (B), but addition of ATX to parietal cells (F) promoted invasion to visceral-like levels. Treatment with S32826 reduced cell invasion in all groups (I-L). Furthermore, both of the Lpar1 inhibitors were efficient at decreasing cell invasion in mesothelial (M, Q) and mesothelioma cells (O, P, S, T). The percent of cell invasion is quantified in panel U. The percent of untreated visceral cells that migrated was set to 100. Asterisks represent statistically significant differences compared to untreated values in each group (p<0.05, n = 4). Error bars were calculated using standard error of the mean.

When migratory behaviors were analyzed on fibronectin substrates, sharp distinction between the two phenotypes was again immediately apparent. Visceral cells had much higher velocity and migrated in a directionally persistent manner as compared to the sedentary parietal cell type (Figure 5 A–B; Figure S1A–B). EMT and subsequent invasive behavior has long been ascribed to the mesothelial cell. To test for potential differences in this activity, Boyden chamber analysis was conducted. As seen in Figure 6, visceral cells were far more invasive than parietal cells, further delineating the more motile/aggressive nature of the visceral cell. The distinct differences in these fundamental properties coupled with the discovery that visceral and parietal mesothelia have vastly different patterns of gene expression suggest that phenotypic variation observed in other basic tissue types extends to mesothelia.

### Autotaxin signaling drives phenotypic diversity in mesothelia

Our data show that autotaxin expression is largely restricted to the more motile and invasive visceral cell type. Given this relationship, we postulated that autotaxin signaling is a critical regulator underlying mesothelial phenotypes.

To explore this signaling pathway further, a chemical genetic screen was conducted to discover novel small molecules that target different points in the autotaxin pathway (Williams, Shelton, Bader, Hong, in preparation). Interestingly, two of the autotaxin pathway inhibitors identified, 2440 and 8437, were found to selectively target Lpar1 (IC_50_  = 1.7 μM, IC_50_  = 6.4 μM, respectively), a G-protein coupled receptor for LPA. Since LPA is a key downstream mediator of autotaxin pathway, this discovery, together with the available direct autotaxin lysophospholipase D inhibitors, expand the list of chemical probes to dissect the roles of the autotaxin pathway in mesothelial biology.

**Figure 7:**
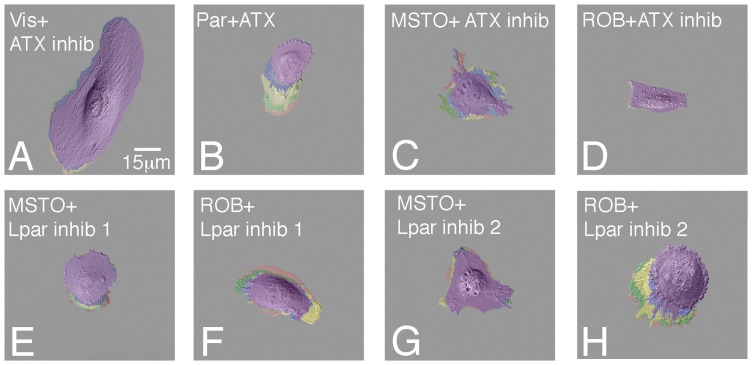
Autotaxin signaling regulates mesothelial and mesothelioma cell migration. Visceral mesothelial, parietal mesothelial, pleural mesothelioma (MSTO), and peritoneal mesothelioma (ROB) cells were plated on fibronectin, cultured with or without the addition of autotaxin, the autotaxin inhibitor S32826, Lpar1 inhibitor 1 (2440), or Lpar1 inhibitor 2 (8437), and subjected to time-lapse imaging in order to visualize their ability to migrate. Panels A-H are compilations of static images taken at 20 minute intervals. False coloration indicates a cell's location at each interval: red 0 min; green 20 min; yellow 40 min; blue 60 min; purple 80 min.

Highly invasive visceral cells were treated with these new compounds and the known autotaxin inhibitor S32826 in Boyden chamber analysis. As seen in Figure 6, this motile mesothelium was highly sensitive to inhibition of autotaxin signaling by S32826 as invasion dropped precipitously with inhibitor application (Figure 6 I, U). Importantly, when cells were treated with the Lpar 1 inhibitors, 2440 or 8437, the same pattern of reduction in cell invasion was observed (Figure 6 M, Q, and U). Conversely, addition of autotaxin to the more sedentary parietal cell stimulated invasion to levels seen with visceral cells (Figure 6F). Linear migration was also sensitive to autotaxin inhibition as a dramatic decrease in motility was observed with S32826 (90% decrease in displacement; Figure 7A, Figure S1), with these cells moving at a rate seen with untreated parietal cells (Figure 5B). In accordance with invasion analyses, addition of autotaxin stimulated parietal cell displacement and velocity to levels seen with inherently motile visceral cells (Figure 7B; Figure S1). Thus, both loss and gain of function analyses demonstrate the importance of this signaling pathway in regulation of mesothelial phenotypes.

While numerous studies have firmly established that visceral mesothelia produce vascular smooth muscle [5], [6], [9], [27], examination of this fundamental property in parietal mesothelia is completely lacking. As presented in Figures S2 and S3, parietal cells also produce smooth muscle cells but at a much reduced rate. Important to the current discussion, use of both Lpar and autotaxin inhibitors blocked muscle differentiation from mesothelia (Figure S2 E–J). Taken together, these data demonstrate that autotaxin signaling is a key regulator of mesothelial phenotypes and that manipulation of this pathway can alter these key mesothelial behaviors through chemical genetic intervention.

### Autotaxin signaling drives mesothelioma phenotypes

Mesotheliomas are a particularly invasive cancer, and autotaxin, which promotes cancer cell invasion, is highly elevated in other types of cancer (21, 28–31). Intriguingly, autotaxin signaling has not been linked to any mesothelioma or manipulated in efforts to subdue their aggressive properties.

When human pleural (MSTO) and peritoneal (ROB) mesotheliomas were probed for activity, exceedingly high levels of autotaxin were observed (Figure 3B). Further and as expected, both mesotheliomas had even greater motility rates, distinct from the two non-cancerous phenotypes (Figure 5 C–D, Figure S1). Interestingly, this heightened migratory behavior was susceptible to inhibition of autotaxin function by S32826 treatment as pleural and peritoneal mesothelioma cell migration was severely inhibited (68% reduction in MSTO displacement, 87% reduction in ROB displacement), reducing motility to that of parietal cells (Figure 7C–D; Figure S1). Similarly, inhibition of Lpar 1 activity also significantly reduced mesothelioma cell migration (Figure 7 E-H; Figure S1). Next, as determined by Boyden analysis, both mesotheliomas were more invasive than the non-cancerous phenotypes (Figure 6 C, D and U). Importantly, this aggressive behavior in both mesotheliomas was also responsive to inhibition of autotaxin and Lpar 1 activity as S32826, 2440 and 8437 were highly effective reducing cellular invasion (Figure 6 K, L, O, P, S, T, U). In summary, autotaxin signaling is intimately linked to migration and invasion of not just mesothelia subtypes but also mesothelioma. Moreover, our data demonstrate that the autotaxin pathway, including autotaxin and the downstream mediator LPA1, is a promising therapeutic target to ameliorate the aggressive characteristics of mesotheliomas.

## Discussion

In this study, we report striking and fundamental differences in cellular behaviors and gene expression profiles between mesothelial cell types. Importantly, our data reveal that autotaxin signaling is an underlying mechanism that regulates divergent cell migration, invasion, and differentiation within mesothelial phenotypes. This finding in turn provides a heretofore-unidentified target to attack in mesotheliomas and pathological conditions involving mesothelia. Additionally, we provide novel reagents to inhibit and/or alter diverse mesothelial behaviors in organogenesis or pathological settings. Taken together, these data advance our fundamental understanding of mesothelial biology and lay the groundwork for future intervention and analysis of mesothelia in development, tissue repair, and disease.

Heterogeneity within the mesothelial phenotype had not been explored. In fact, until now, all mesothelia have been regarded as a single cell type “hardly varying from site to site” [15]. Here, in examining the most basic division of mesothelia, visceral vs. parietal types, fundamental differences between these cell types were readily apparent. These seemingly simple results provide a conceptual framework for the future exploration of the divergent functions and pathological outcomes seen in mesothelia. For example, abdominal adhesions are characterized by mesothelial attachments between adjacent organs and the body wall [28]. However, little is known regarding the molecular cues governing the transition from normal mesothelial cell to pathological cells having the potential to form patches of fibrous and vascularized tissue, [28]. With the understanding that adhesive, motile, invasive, and vasculogenic behaviors are elevated in visceral mesothelium, interventional therapies may target this cell type in an effort in mitigate mesothelial cell contribution to post-operative adhesive scars.

The discovery that levels of autotaxin signaling are predictive of a mesothelial cell's migratory and invasive behavior is of particular interest. First, the highly restricted expression of this secreted lysophospholipase D initially discovered as a tumor cell-motility-stimulating factor provides mechanistic insight into the regulation of mesothelial cell behaviors [24]. Elevated levels of autotaxin in visceral mesothelium align with the more adhesive, motile, and invasive traits of this phenotype compared to its sedentary parietal counterpart. It is also important to note that alteration of autotaxin levels and the subsequent, predictable changes in behaviors of either visceral or parietal mesothelia reveals innate plasticity within this tissue type. This is reminiscent of other basic tissues including skeletal muscle where alterations in workload, intracellular calcium levels, and frequency of use lead to distinct changes in cell physiology [29], [30], [31]. The malleable nature of mesothelial cells may help to explain why these cells can play such diverse roles in normal development, homeostasis, tissue repair, and disease.

Here for the first time, we demonstrate that autotaxin is found at abnormally high levels in human pleural and peritoneal mesotheliomas and that this signaling pathway regulates migratory and invasive of these cancerous cells. Autotaxin has also been implicated in chronic inflammatory disorders, diabetes, arthritis, and atherosclerosis [32], [33], [34]. Given its role in human disease, the autotaxin-LPA axis has been attractive as a diagnostic marker and therapeutic target [19]. An aggressive form of cancer, mesothelioma is generally unresponsive to conventional chemotherapeutic and radiation therapies [12]. Thus, discovery of autotaxin regulation of mesothelioma migration and invasion and their responsiveness to inhibitors of this pathway provide a viable target in mitigating cancer progression. Our newly discovered Lpar1 inhibitors intervene at a novel point in the autotaxin signaling pathway presenting an opportunity for “combinatorial” intervention in combating this aggressive cancer.

## Methods

### Animal Procedures

All animal procedures were approved and performed in accordance with Vanderbilt University's Division of Animal Care and IACUC/OAWA.

### Immunohistochemistry

Small intestine, omentum, and body wall tissue samples were fixed in 4% paraformaldehyde for 2 h prior to sucrose infiltration and cryo-sectioning. Cultured cells were fixed in 4% paraformaldehyde for 30 min prior to staining. Blocking solution (10% goat serum, 0.25% Triton X-100 in PBS) was applied for 1 h at room temperature. Primary and secondary antibodies are listed in Table S2. Samples containing primary antibodies were incubated overnight at 4°C. Samples containing secondary antibodies and DAPI were incubated for 3 h at room temperature.

### Mesothelial Cell Isolation and Culture

Visceral mesothelium was isolated from the omentum of adult Wt1-cre; Rosa26R^eYFP^ mice as previously reported [27] (Figure S4A). In parallel, parietal mesothelium was isolated by teasing the mesothelium from the underlying skeletal muscle of the body wall of these animals (Figure S4B). RNA was then extracted from these native mesothelia samples and used for hybridization in a microarray experiment. In addition, tissue isolated in this way was dissociated into single cell suspensions using collagenase (Sigma, C2799) and cultured on fibronectin-coated glass Lab-Tek chamber slides in supplemented medium (10% fetal bovine serum, 1% penicillin/streptomycin, Dulbecco's modified Eagle's medium) as previously reported [9] (Figure S4C, D). MSTO-211H human pleural mesothelioma cells (ATCC, CRL-2081) and ROB human peritoneal mesothelioma cells [35] (a kind gift from Raffit Hassan, MD, National Cancer Institute, National Institutes of Health) were also cultured in supplemented medium and plated on fibronectin-coated glass chamber slides. Some cultured cells were treated with 10 nM Autotaxin (Echelon, E-4000), 1 µM of the autotaxin inhibitor, S32826, (Sigma, S1825), 1 µM of the Lpar1 inhibitors, 2440 (IC_50_  = 1.7 µM) and 8437 (IC_50_  = 6.4 µM).

### Collagen Gel Contraction Assay

Visceral and parietal mesothelial cells were suspended (500,000cells/mL) in a rat tail collagen 1/PBS solution (2.2 mg/mL collagen; 1.8 mg/mL NaHCO_3_) [36]. 250 ml of cell suspension was aliquoted into a single well of a 24-well plate and placed at 37°C for 45 minutes to allow for collagen polymerization. Then collagen gels were then detached from the walls using a pipet tip and 250 ml of supplemented media was added to each well. The gels were incubated at 37°C for 7 days after which the area of each gel was determined and compared to the area of a well.

### Quantitative Real Time RT-PCR

RNA was isolated using Trizol. 100 g cDNA template was generated using a High Capacity cDNA Reverse Transcription Kit (Applied Biosystems). Relative levels of gene expression were determined using TaqMan-based quantitative RT–PCR on a 7900HT platform. The TaqMan Gene Expression Assay (Applied Biosystems) for Autotaxin (Mm00516572_m1) was used. 18S (Hs99999901_s1) was used as an internal control. Triplicate DCT values were generated for each assay. The fold change in expression was determined by dividing experimental values by the control value, which was then set to 1.

### Migration Live Cell Imaging

For each cell type, DIC images were recorded every minute for 2 h using a DeltaVision imaging system and softWoRx image acquisition software (Applied Precision). False colored images were generated for the position of each cell at 20 minute intervals. Cell migration tracks were quantified using the manual tracking plug-in of ImageJ. The position of each nucleus in DIC recordings was used as reference points. Displacement values were quantified as the total migration track. Velocity values were calculated by taking the displacement value divided by time (80 minutes). Directional persistence values were quantified as the final distance of cell relocation divided by total migration track.

### Microarray Analysis

Whole transcriptome expression analysis of total RNA was performed using Affymetrix mouse Gene 1.0 ST arrays, which cover 26,166 RefSeq transcripts. RNA quality was assessed using an Agilent 2100 before further processing and hybridization in the Vanderbilt Functional Shared Resource (http://www.thefgsr.com/). Microarray images were scanned with an Affymetrix high resolution GenePix 4000B scanner. Raw.CEL files were subsequently uploaded into Partek Genomics Suite version 6.6 (Partek Incorporated, St. Louis, MO) and processed using Robust Multi-chip Average (RMA) normalization [37], [38]. Following RMA normalization, Partek was used to perform pairwise comparisons of average group values and one-way ANOVA with Benjamini & Hochberg (B-H) multiple hypothesis correction for analysis of visceral and parietal tissues. Only probes that resulted in a fold-difference of at least 1.5 and B-H corrected *p* value of less than 0.05 were considered significantly altered. Statistical analyses (including B-H correction for multiple hypothesis testing) for identification of overrepresented ontologies, functions, and pathways were performed using DAVID (http://david.abcc.ncifcrf.gov), after initial statistical data analysis was performed to identify relevant gene sets. Using DAVID, genes were grouped based on functional similarity. All microarray data has been deposited in the Gene Expression Omnibus database, accession number GSE47161.

### Autotaxin Activity Assay

Visceral mesothelial, parietal mesothelial, MSTO, and ROB cells were cultured in serum free medium for 48 h. Conditioned media from these cells was collected and used in a commercially available autotaxin activity assay (Echelon, K-4100). ATX activity in the media was analyzed using the fluorogenic substrate FS-3 according to the manufacture protocol [39]. Briefly, 50 ml of each sample was mixed with 5 mM FS-3 and assayed in 96-well plate. The change of fluorescent intensity was measured by a Multimode Plate Reader Synergy HT (BioTek) with excitation and emission wavelengths of 485 and 528 nm respectively. Readings were taken every 2 minutes for 2 h. The amount of fluorescence is expressed as an ATX unit, which is defined as pM FS-3 hydrolyzed/min in 10 μM FS-3, 50 mM Tris-Cl pH 8.0, 5 mM KCl,1 mM CaCl2, 1 mM MgCl2, 140 mM NaCl, 1 mg/ml Fatty Acid Free BSA, 1 mM LPC at 37°C.

### Boyden Chamber Invasion Assay

Cell invasion was measured as previously reported [9]. Briefly, single cell suspensions were resuspended in supplemented media at a density of 1× 10^5^ cells/mL. Each cell suspension was added to the top chamber of a Boyden chamber culture plate insert (8 µm pores; Millicell) and placed in a 24-well culture plate containing supplemented media with or without ATX or one of the inhibitors. Cells were incubated for 6 h. Cells that migrated through the pores and were adherent to the bottom of the insert were fixed in 100%MeOH and stained with Giemsa (Sigma-Aldrich). The percent of cell migration was calculated by dividing the number of migrated cells in each group by the number of migrated cells in the visceral mesothelium group and multiplying by 100. The percent of untreated visceral cells that migrated was set to 100.

## Supporting Information

Figure S1 **Autotaxin signaling regulates mesothelial and mesothelioma cell migration.** Visceral mesothelial, parietal mesothelial, pleural mesothelioma (MSTO), and peritoneal mesothelioma (ROB) cells were cultured with and without the addition of autotaxin (ATX), the autotaxin inhibitor S32826, Lpar1 inhibitor 1 (2440), or Lpar1 inhibitor 2 (8437) and subjected to time-lapse imaging to monitor cell migration. The displacement (A) and velocity (B) for each cell type was calculated and quantified. The displacement and velocity of untreated visceral cells was set to 1 and fold changes were calculated for each subsequent cell type. Error bars were calculated using standard error of the mean (n = 15).
(TIF)

Figure S2 **Autotaxin signaling regulates smooth muscle differentiation in mesothelia.** Visceral and parietal mesothelial cells were cultured for 3 days with or without addition of autotaxin (ATX), the autotaxin inhibitor S32826, Lpar1 inhibitor 1 (2440), or Lpar1 inhibitor 2 (8437). Cells were analyzed for the expression of the smooth muscle α-actin (αsma) and smooth muscle myosin heavy chain (smMHC) by immunofluorescence. Visceral cells readily differentiated into smooth muscle and expressed both markers (A). Addition of ATX to visceral cells further promoted smooth muscle differentiation and increased the number of cells staining positive for smMHC (C), while addition of the S32826 (E) or the Lpar1 inhibitors (G, I) significantly decreased the number of smooth muscle cells. In contrast, untreated parietal cells rarely expressed αsma and were devoid of smMHC expression (B). However, addition of ATX to parietal cells promoted smooth muscle marker expression (D). Addition of S32826 or the Lpar1 inhibitors to parietal cells extinguished both smooth muscle markers (F, H, J). Nuclei are marked with DAPI.
(TIF)

Figure S3 **Visceral mesothelial cells are more contractile compared to parietal mesothelial cells.** Visceral and parietal mesothelial cells were seeded in collagen gel and cultured for 8 days. The area of each gel (white outline) was measured and compared to its original area (green outline). The visceral gel (A) was significantly smaller than the parietal gel (B). Areas are quantified in panel C. The asterisk represents a statistically significant difference (p<0.05, n = 5). Error bars were calculated using standard error of the mean.
(TIF)

Figure S4 **Isolation of visceral and parietal mesothelia.** Visceral and parietal mesothelia were isolated from omentum (A) and the body wall (B) of adult Wt1-cre; Rosa26R^eYFP^ mice. Cells were dissociated, cultured for 7 days, and stained for the eYFP marker to indicate a pure population of isolated cells.
(TIF)

Table S1 **Examples of genes differentially expressed in visceral and parietal mesothelia.** Positive fold differences indicate higher expression in visceral mesothelium and negative fold differences indicate lower expression in visceral mesothelium compared to parietal mesothelium.
(DOCX)

Table S2 **Primary and Secondary Antibodies.**
(DOC)
