# Supplementary figures and images for: Autotaxin Signaling Governs Phenotypic Heterogeneity in Visceral and Parietal Mesothelia
Source: PLoS One. 2013 Jul 25;8(7):e69712. doi: 10.1371/journal.pone.0069712 (PMC3723636; doi:10.1371/journal.pone.0069712)

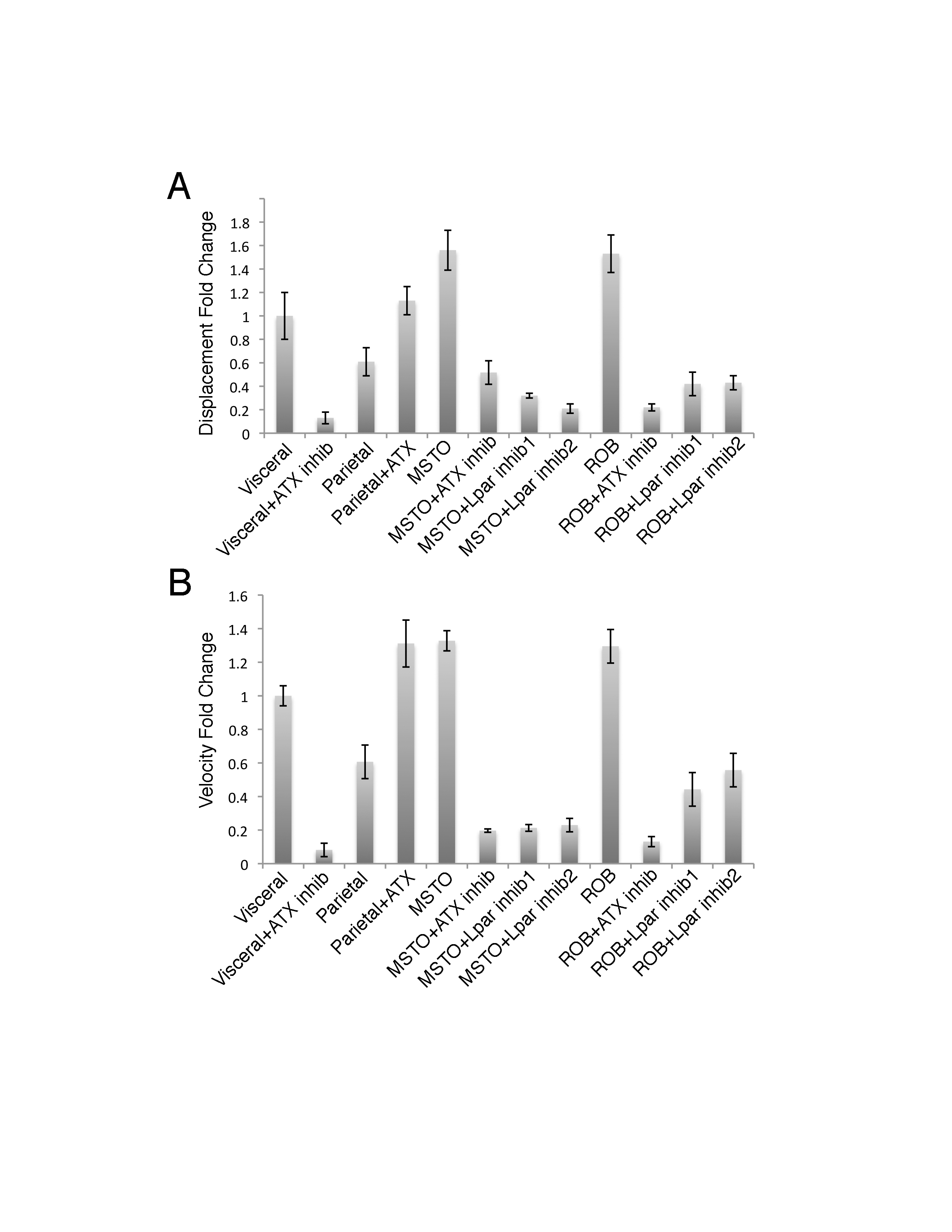

Supplement: Figure S1 — Autotaxin signaling regulates mesothelial and mesothelioma cell migration. Visceral mesothelial, parietal mesothelial, pleural mesothelioma (MSTO), and peritoneal mesothelioma (ROB) cells were cultured with and without the addition of autotaxin (ATX), the autotaxin inhibitor S32826, Lpar1 inhibitor 1 (2440), or Lpar1 inhibitor 2 (8437) and subjected to time-lapse imaging to monitor cell migration. The displacement (A) and velocity (B) for each cell type was calculated and quantified. The displacement and velocity of untreated visceral cells was set to 1 and fold changes were calculated for each subsequent cell type. Error bars were calculated using standard error of the mean (n = 15). (TIF) [file pone.0069712.s001.tif]

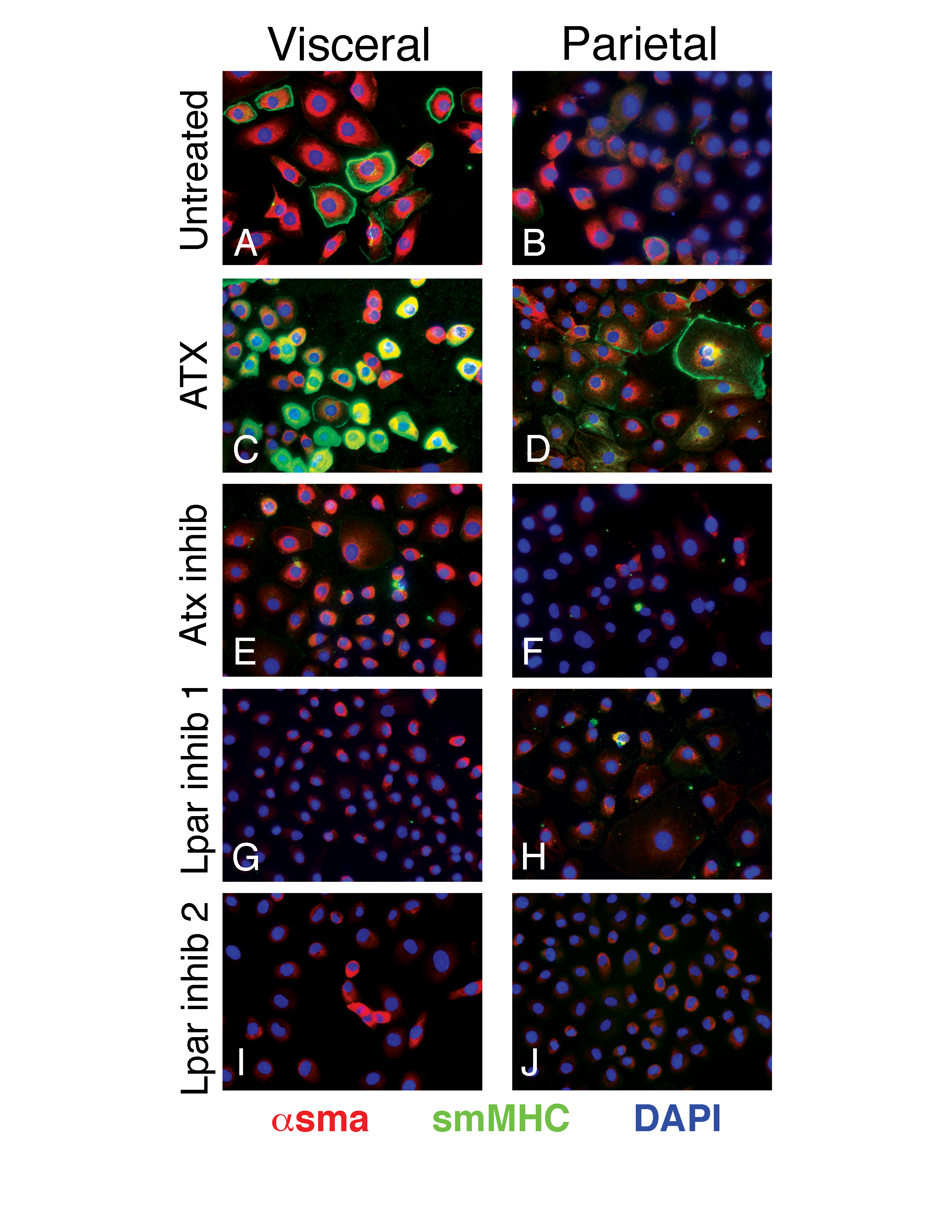

Supplement: Figure S2 — Autotaxin signaling regulates smooth muscle differentiation in mesothelia. Visceral and parietal mesothelial cells were cultured for 3 days with or without addition of autotaxin (ATX), the autotaxin inhibitor S32826, Lpar1 inhibitor 1 (2440), or Lpar1 inhibitor 2 (8437). Cells were analyzed for the expression of the smooth muscle α-actin (αsma) and smooth muscle myosin heavy chain (smMHC) by immunofluorescence. Visceral cells readily differentiated into smooth muscle and expressed both markers (A). Addition of ATX to visceral cells further promoted smooth muscle differentiation and increased the number of cells staining positive for smMHC (C), while addition of the S32826 (E) or the Lpar1 inhibitors (G, I) significantly decreased the number of smooth muscle cells. In contrast, untreated parietal cells rarely expressed αsma and were devoid of smMHC expression (B). However, addition of ATX to parietal cells promoted smooth muscle marker expression (D). Addition of S32826 or the Lpar1 inhibitors to parietal cells extinguished both smooth muscle markers (F, H, J). Nuclei are marked with DAPI. (TIF) [file pone.0069712.s002.tif]

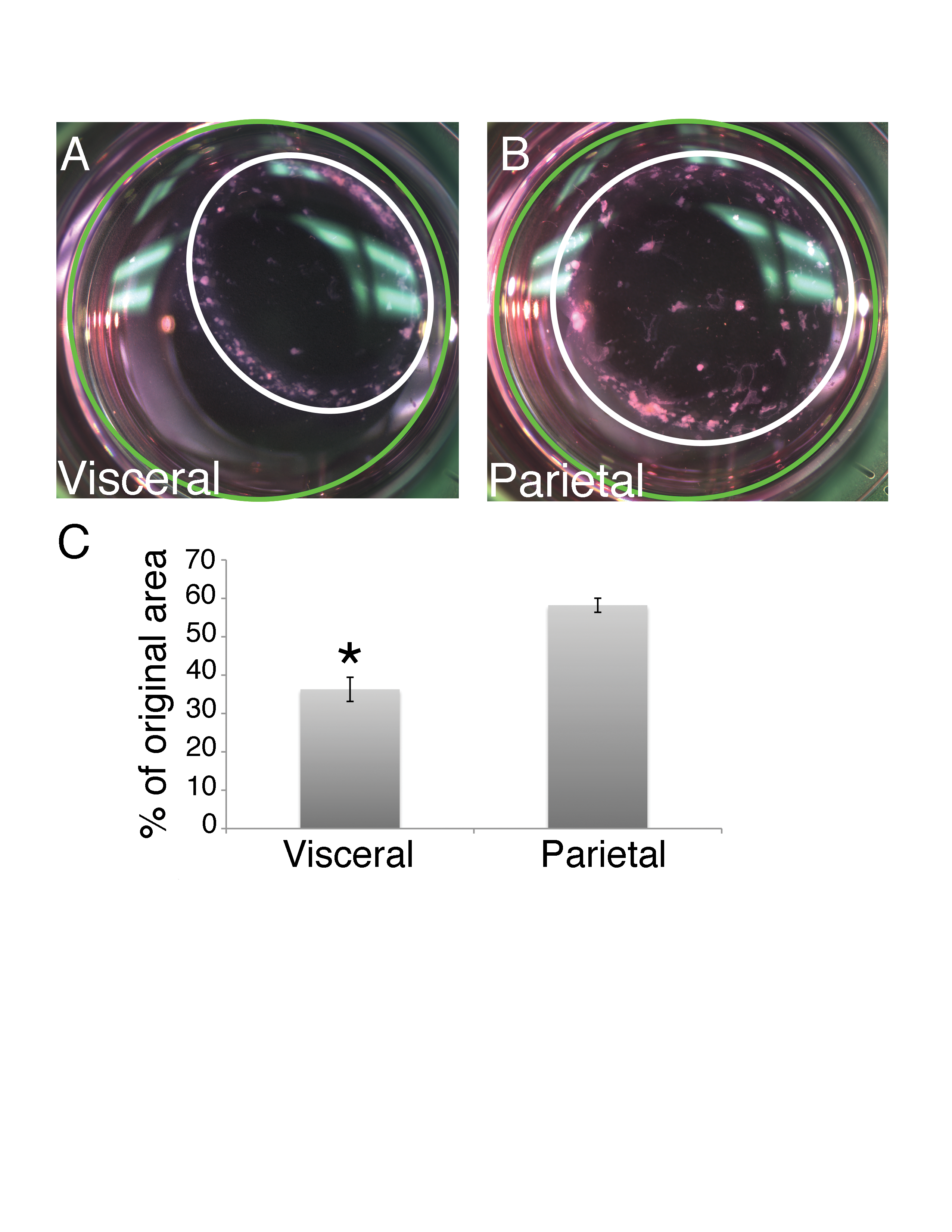

Supplement: Figure S3 — Visceral mesothelial cells are more contractile compared to parietal mesothelial cells. Visceral and parietal mesothelial cells were seeded in collagen gel and cultured for 8 days. The area of each gel (white outline) was measured and compared to its original area (green outline). The visceral gel (A) was significantly smaller than the parietal gel (B). Areas are quantified in panel C. The asterisk represents a statistically significant difference (p<0.05, n = 5). Error bars were calculated using standard error of the mean. (TIF) [file pone.0069712.s003.tif]

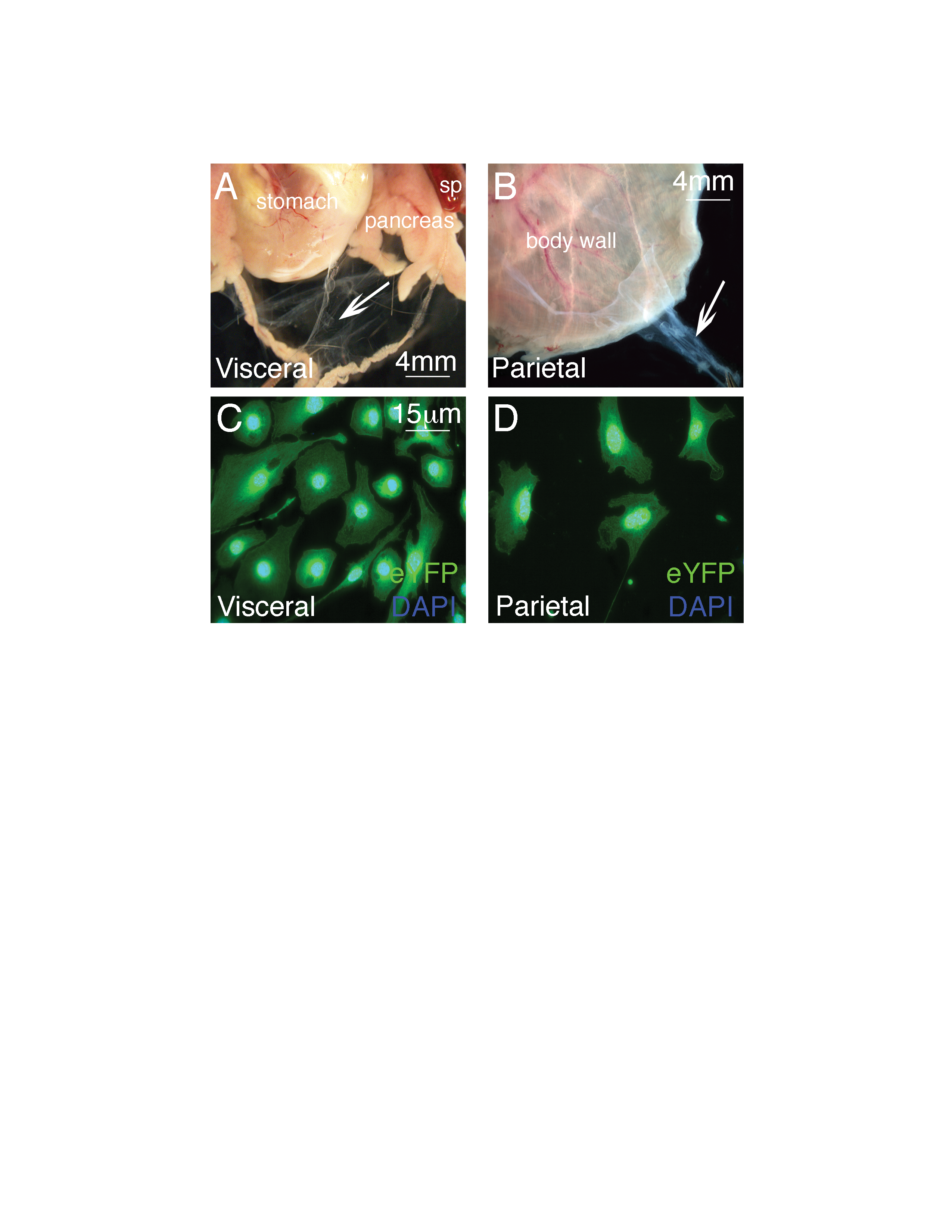

Supplement: Figure S4 — Isolation of visceral and parietal mesothelia. Visceral and parietal mesothelia were isolated from omentum (A) and the body wall (B) of adult Wt1-cre; Rosa26ReYFP mice. Cells were dissociated, cultured for 7 days, and stained for the eYFP marker to indicate a pure population of isolated cells. (TIF) [file pone.0069712.s004.tif]
