# Supplementary material for: Autotaxin Signaling Governs Phenotypic Heterogeneity in Visceral and Parietal Mesothelia
Source: PLoS One. 2013 Jul 25;8(7):e69712. doi: 10.1371/journal.pone.0069712 (PMC3723636; doi:10.1371/journal.pone.0069712)
Supplement: Table S1 — Examples of genes differentially expressed in visceral and parietal mesothelia. Positive fold differences indicate higher expression in visceral mesothelium and negative fold differences indicate lower expression in visceral mesothelium compared to parietal mesothelium. (DOCX) [file pone.0069712.s005.docx]

| **Gene Symbol** | **Gene Name** | **Visceral/Pariental** | |
| --- | --- | --- | --- |
|  | | **p-value** | **Fold difference** |
| Adamts15 | a disintegrin-like and metallopeptidase (reprolysin type) with thrombospondin type 1 motif, 15 | 3.7E-02 | 1.8 |
| Adamts16 | a disintegrin-like and metallopeptidase (reprolysin type) with thrombospondin type 1 motif, 16 | 3.5E-02 | 2.4 |
| Adamts19 | a disintegrin-like and metallopeptidase (reprolysin type) with thrombospondin type 1 motif, 19 | 3.7E-02 | 2.9 |
| Adamts2 | a disintegrin-like and metallopeptidase (reprolysin type) with thrombospondin type 1 motif, 2 | 1.1E-02 | -1.8 |
| Adamts3 | a disintegrin-like and metallopeptidase (reprolysin type) with thrombospondin type 1 motif, 3 | 3.5E-02 | 1.9 |
| Adamts8 | a disintegrin-like and metallopeptidase (reprolysin type) with thrombospondin type 1 motif, 8 | 1.2E-02 | 2.6 |
| Adamts9 | a disintegrin-like and metallopeptidase (reprolysin type) with thrombospondin type 1 motif, 9 | 4.6E-02 | 1.9 |
| Add3 | adducin 3 (gamma) | 1.3E-03 | 3.1 |
| Alcam | activated leukocyte cell adhesion molecule | 2.2E-03 | 2.0 |
| Amigo2 | adhesion molecule with Ig like domain 2 /// family with sequence similarity 113, member B | 1.4E-02 | -1.8 |
| Atx | autotaxin | 6.9E-04 | 48.4 |
| Bcam | basal cell adhesion molecule | 1.8E-03 | -3.6 |
| Boc | biregional cell adhesion molecule-related/down-regulated by oncogenes (Cdon) binding protein | 2.8E-03 | 2.7 |
| Bves | blood vessel epicardial substance | 1.9E-02 | -3.9 |
| Cadm3 | cell adhesion molecule 3 | 1.3E-02 | 4.1 |
| Cdh13 | cadherin 13 | 4.8E-03 | -2.2 |
| Cdh3 | cadherin 3 | 1.1E-04 | -10.2 |
| Cdh4 | cadherin 4 | 9.6E-03 | -1.6 |
| Clec7a | C-type lectin domain family 7, member a | 1.6E-02 | 3.4 |
| Cntn1 | contactin 1 | 3.1E-03 | -1.8 |
| Col14a1 | collagen, type XIV, alpha 1 | 6.6E-03 | -6.0 |
| Col1a2 | collagen, type I, alpha 2 | 4.2E-02 | -2.0 |
| Col4a4 | collagen, type IV, alpha 4 | 8.5E-03 | -1.6 |
| Col8a1 | collagen, type VIII, alpha 1 | 2.1E-02 | -7.4 |
| Col8a1 | collagen, type VIII, alpha 1 | 2.1E-02 | -7.4 |
| Cytip | cytohesin 1 interacting protein | 1.9E-02 | 2.0 |
| Dnm1 | dynamin 1 | 2.0E-02 | 1.8 |
| Eln | elastin | 1.5E-02 | -5.1 |
| Emb | embigin | 1.1E-02 | 10.2 |
| Emilin2 | elastin microfibril interfacer 2 | 5.7E-03 | 5.1 |
| Esam | endothelial cell-specific adhesion molecule | 3.9E-03 | -1.6 |
| Fbln1 | fibulin 1 | 8.1E-04 | 1.7 |
| Fbln5 | fibulin 5 | 3.1E-03 | -2.1 |
| Fn1 | fibronectin 1 | 7.2E-03 | 3.7 |
| Gja1 | gap junction protein, alpha 1 | 6.8E-03 | -3.0 |
| Gpc1 | glypican 1 | 1.1E-03 | -1.8 |
| Gpc3 | glypican 3 | 1.2E-02 | -1.5 |
| Icam1 | intercellular adhesion molecule 1 | 1.7E-02 | -2.0 |
| Itga2 | integrin alpha 2 | 2.9E-02 | -2.0 |
| Itga3 | integrin alpha 3 | 9.9E-03 | -1.7 |
| Itga4 | integrin alpha 4 | 6.6E-03 | 2.3 |
| Itgam | integrin alpha M | 2.4E-03 | 3.1 |
| Itgb1bp2 | integrin beta 1 binding protein 2 | 2.6E-02 | -1.8 |
| Itgb2 | integrin beta 2 | 5.1E-03 | 3.4 |
| Itgb6 | integrin beta 6 | 2.5E-02 | -2.2 |
| Itgbl1 | integrin, beta-like 1 | 9.8E-03 | -15.8 |
| Jam3 | junction adhesion molecule 3 | 2.5E-03 | 2.5 |
| Kifc3 | kinesin family member C3 | 8.7E-03 | -1.7 |
| Kit | kit oncogene | 2.9E-02 | 3.5 |
| Lama2 | laminin, alpha 2 | 1.2E-03 | 2.5 |
| Lama5 | laminin, alpha 5 | 5.6E-03 | -1.8 |
| Lamc3 | laminin gamma 3 | 8.5E-03 | 3.6 |
| Lcp1 | lymphocyte cytosolic protein 1 | 1.3E-03 | 6.3 |
| Lgals1 | lectin, galactose binding, soluble 1 | 1.0E-02 | 2.0 |
| Lgals3 | lectin, galactose binding, soluble 3 | 5.2E-03 | -2.2 |
| Lmo7 | LIM domain only 7 | 1.6E-03 | -6.6 |
| Mmp16 | matrix metallopeptidase 16 | 1.4E-02 | 2.4 |
| Mmp19 | matrix metallopeptidase 19 | 3.6E-02 | 3.0 |
| Mmp9 | matrix metallopeptidase 9 | 1.3E-02 | 1.8 |
| Mpzl2 | myelin protein zero-like 2 | 3.3E-03 | -1.8 |
| Msln | mesothelin | 1.8E-03 | -4.8 |
| Ncam1 | neural cell adhesion molecule 1 | 2.0E-02 | -2.9 |
| Nrp1 | neuropilin 1 | 1.4E-02 | 2.1 |
| Ntn4 | netrin 4 | 2.9E-02 | -2.0 |
| Ntng2 | netrin G2 | 5.9E-03 | 1.7 |
| Olfml2a | olfactomedin-like 2A | 2.5E-02 | 3.2 |
| Olfml2b | olfactomedin-like 2B | 3.2E-02 | 2.4 |
| Pcdh15 | Protocadherin 15 | 1.6E-02 | 3.3 |
| Pcdh18 | Protocadherin 18 | 3.8E-02 | 2.2 |
| Pcdh9 | protocadherin 9 | 4.4E-02 | 1.8 |
| Pkp2 | plakophilin 2 | 3.3E-02 | -1.7 |
| Plat | plasminogen activator, tissue | 1.1E-04 | 4.2 |
| Plau | plasminogen activator, urokinase | 6.8E-03 | 4.5 |
| Plek | pleckstrin | 1.6E-03 | 3.0 |
| Plek2 | pleckstrin 2 | 1.7E-02 | 1.8 |
| Plekhh2 | pleckstrin homology domain containing, family H (with MyTH4 domain) member 2 | 1.0E-03 | -1.7 |
| Sell | selectin, lymphocyte | 8.6E-03 | 1.8 |
| Selplg | selectin, platelet (p-selectin) ligand | 2.9E-02 | 2.9 |
| Synm | synemin, intermediate filament protein | 1.2E-02 | -1.9 |
| Synpo | synaptopodin | 1.8E-02 | -1.9 |
| Synpo2l | synaptopodin 2-like | 3.2E-02 | -2.0 |
| Timp1 | tissue inhibitor of metalloproteinase 1 | 4.0E-03 | -5.4 |
| Tln2 | talin 2 | 6.0E-04 | -1.9 |
| Vcam1 | vascular cell adhesion molecule 1 | 1.2E-02 | 1.8 |
| Vit | vitrin | 1.6E-03 | 12.6 |
| Vnn1 | vanin 1 | 2.1E-04 | -8.3 |
