# Supplementary material for: Autotaxin Signaling Governs Phenotypic Heterogeneity in Visceral and Parietal Mesothelia
Source: PLoS One. 2013 Jul 25;8(7):e69712. doi: 10.1371/journal.pone.0069712 (PMC3723636; doi:10.1371/journal.pone.0069712)
Supplement: Table S2 — Primary and Secondary Antibodies. (DOC) [file pone.0069712.s006.doc]

| **Antibody** | **Source** | **Dilution** |
| --- | --- | --- |
| GFP | Abcam, ab1218 | 1:250 |
| Autotaxin | Santa Cruz Biotechnology, sc-66813 | 1:100 |
| αsma | Sigma, A 2547 | 1:250 |
| smMHC | Biomedical Technologies Inc., BT-562 | 1:200 |
| vinculin | Abcam, ab18058 | 1:200 |
| phalloidin | Invitrogen, A12380 | 1:100 |
| Alexa Fluor 488 goat anti-rabbit IgG | Invitrogen, A-11008 | 1:2000 |
| Alexa Fluor 568 goat anti-rabbit IgG | Invitrogen, A-11011 | 1:2000 |
| Alexa Fluor 568 goat anti-mouse IgG | Invitrogen, A-11004 | 1:2000 |
| Alexa Fluor 488 goat anti-mouse IgG | Invitrogen, A-11001 | 1:2000 |

**Table S2. Primary and Secondary antibodies**
